# A novel long noncoding RNA, TMEM92‐AS1, promotes gastric cancer progression by binding to YBX1 to mediate CCL5

**DOI:** 10.1002/1878-0261.12863

**Published:** 2021-01-12

**Authors:** Shubin Song, Xuezhi He, Jing Wang, Hongtao Song, Yimin Wang, Yansong Liu, Zhengbo Zhou, Zhiyong Yu, Dengshun Miao, Yingwei Xue

**Affiliations:** ^1^ Department of Gastrointestinal Surgery Harbin Medical University Cancer Hospital China; ^2^ Department of Breast Surgery Shandong Cancer Hospital and Institute Shandong First Medical University and Shandong Academy of Medical Sciences Jinan China; ^3^ Department of Anatomy, Histology and Embryology The Research Centre for Bone and Stem Cells Nanjing Medical University China; ^4^ Department of Anatomy, Histology and Embryology State Key Laboratory of Reproductive Medicine The Research Centre for Bone and Stem Cells Nanjing Medical University China; ^5^ Department of Pathology Harbin Medical University Cancer Hospital China; ^6^ The Research Centre for Ageing Friendship Affiliated Plastic Surgery Hospital of Nanjing Medical University China

**Keywords:** CCL5, gastric cancer, lncRNA, YBX1

## Abstract

Numerous studies have revealed that long noncoding RNAs (lncRNAs) with oncogene properties play vital roles in gastric cancer (GC). In this study, we aimed to elucidate the function of TMEM92‐AS1 in GC progression and to investigate its underlying mechanisms. TMEM92‐AS1 was filtered from the Gene Expression Omnibus database. GC tissues and adjacent normal tissues were used to detect the expression level of TMEM92‐AS1. MTT, colony‐formation assays, Edu, cell cycle, apoptosis and subcutaneous tumour formation assays were used to detect the role of TMEM92‐AS1 in cell function. RNA transcriptome sequencing was used to seek downstream target genes. Reverse transcription (RT)‐qPCR, western blot, RNA and chromatin immunoprecipitation assays were used to investigate the mechanisms involved. TMEM92‐AS1 was significantly overexpressed in GC tissues and correlated with poor overall survival and disease‐free survival. Furthermore, TMEM92‐AS1 promoted GC cell proliferation and migration *in vitro* and tumorigenic ability *in vivo*. RNA transcriptome sequence analysis revealed a potential downstream target gene, C‐C motif chemokine ligand 5 (CCL5), and a mechanistic study found that TMEM92‐AS1 regulated CCL5 by binding to the transcription factor Y‐box binding protein 1(YBX1), which has oncogene properties. In addition, TMEM92‐AS1 was found to be associated with peripheral blood leukocyte counts, especially neutrophils. Further investigation found that TMEM92‐AS1 may affect leukocytes via regulation of the expression of granulocyte colony‐stimulating factor in GC tissues. Our data provide an in‐depth insight into the mechanism behind the lncRNA TMEM92‐AS1, how it promotes GC progression and the possible mechanism in affecting peripheral leukocyte counts. Therefore, TMEM92‐AS1 is a potential target for GC individualized therapy and prognostic assessment.

## Graphical abstract

Oncogene‐like effects of TMEM92‐AS1: LncRNA TMEM92‐AS1 regulates the expression of downstream target gene CCL5 by binding with YBX1 protein to play the role of oncogene and affects prognosis of patients with gastric cancer. TMEM92‐AS1 may also promote the secretion of G‐CSF by tumour cells to increase the level of neutrophils in the peripheral blood.

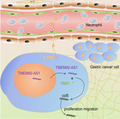

**Abbreviations**
CCL5: C‐C motif chemokine ligand 5
CXCL2: C‐X‐C motif chemokine ligand 2
EMT: epithelial‐mesenchymal transition
GC: gastric cancer
G‐CSF: granulocyte colony‐stimulating factor
IL‐6: interleukin‐6
KDM4A: lysine demethylase 4A
lncRNA: long noncoding RNA
MLNR: metastatic lymph node rate
NLR: neutrophil to lymphocyte ratio
NWR: neutrophil to white blood cell ratio
RBP: RNA‐binding protein
YBX1: Y‐box binding protein 1

## 1 Introduction

The Human Genome Research Program found that the transcription of human genomes produces large amounts of noncoding RNA (ncRNA), especially transcripts longer than 200 bp, which we now call long noncoding RNA (lncRNA) [1]. Although lncRNA do not have the ability to encode proteins, or only encode very short peptide chains, they participate in a wide range of cellular functions, such as cell proliferation, differentiation, apoptosis, invasion and migration [2, 3, 4]. Unlike messenger RNA (mRNA) that are primarily located in the cytoplasm and are used to synthesize proteins, lncRNA are located either in the nucleus or in the cytoplasm, which allows them to perform a variety of functions in different cellular substructures [5].

Tumour development, progression and metastasis are processes involving multiple genetic or epigenetic changes, leading to oncogene activation or tumour suppressor gene inactivation. Gastric cancer (GC), one of the most common malignant tumours of the digestive system, often leads to a poor prognosis in patients due to late detection and early metastasis. In the past, research on gastric cancer focused on genes that encode proteins because these genes are simple in structure, clear in regulation, and the main components of cellular function.

With the deepening of studies on the structure and function of genes, increasing numbers of discoveries have documented the important role of lncRNA in tumours. For example, the lncRNA ATB is involved in cell functions, and its high expression predicts a poor outcome of colorectal cancer [6]. The lncRNA UCA1 is an oncogene and mediates the expression of ERBB4 [7]. Upregulation of the lncRNA HOTAIR promotes the proliferation of breast cancer cells and contributes to their resistance to tamoxifen [8]. Although we have identified a large number of functions of lncRNA over the years, we still have a long way to go fully to understand the pathogenesis and mechanisms of lncRNA in affecting tumours.

Considering the gap in lncRNA research, we conducted this study to explore the expression alteration of a specific lncRNA, TMEM92‐AS1, which is located on 17q21.33, to identify its prognostic significance and mechanisms in gastric cancer. In the present study, we confirmed the oncogenic role of TMEM92‐AS1 and found that it shows promise as a prognostic biomarker in GC patients. We further found that knockdown of TMEM92‐AS1 inhibited the proliferation and migration of GC cells. Moreover, mechanistic studies found that TMEM92‐AS1 functions, at least in part, by binding to the Y‐box binding protein 1 (YBX1) protein to regulate the expression of the downstream target gene CCL5.

## 2 Materials and methods

### 2.1 Tissue collection and ethics statement

Gastric cancer specimens and adjacent normal tissues were obtained from 108 GC patients who received surgery at the Harbin Medical University Cancer Hospital. None of these patients received a blood transfusion or anticancer therapy and all were without infectious disease before surgery. All of the tissues were sectioned immediately after leaving the body, frozen in liquid nitrogen and stored at −80 °C until use. All patients were followed up at least once every 3 months during the first 2 years after surgery and every 1 year after the first 2 years. The patient’s clinical pathology characteristics were collected from their medical records. Overall survival (OS) was defined as the time from surgery to death, and disease‐free survival (DFS) was defined as the time from surgery to GC recurrence or progression. This study was approved by the Medical Ethics Committee of the Harbin Medical University Cancer Hospital, and all of the patients provided informed consent to allow the use of their data for any future study. The experiments were undertaken with the understanding and written consent of each subject. The study methodologies conformed to the standards set by the Declaration of Helsinki.

### 2.2 RNA extraction and qRT‐PCR analyses

Total RNA extraction from tissues or cells was conducted using TRIzol reagent (Life Technologies, Carlsbad, CA, USA). RNA was reverse transcribed into cDNA using a Reverse Transcription Kit (Takara, Dalian, China). Quantitative reverse transcriptase polymerase chain reaction (qRT‐PCR) was carried out using SYBR Green (Takara). The samples were subjected to an initial single cycle of 5 min at 95 °C, followed by 10 s at 95 °C, and 30 s at 60 °C for 40 cycles. Glyceraldehyde‐3‐phosphate dehydrogenase (GAPDH) was used as an internal reference. Relative quantification of differential mRNA expression was calculated using the $2^{‐\Delta \Delta C_{T}}$ method relative to GAPDH. The primers used are listed in Table S1.

### 2.3 Cell culture

Human GC cell lines (BGC823, SGC7901, MGC803, HGC27 and MKN45) and the normal gastric epithelial cell line GES1 were purchased from the Institute of Biochemistry and Cell Biology of the Chinese Academy of Sciences (Shanghai, China). Cells were cultured in RPMI 1640 or DMEM (GIBCO‐BRL, ThermoFisher Scientific, Carlsbad, CA, USA) medium supplemented with 10% FBS, 100 U·mL^−1^ penicillin and 100 mg·mL^−1^ streptomycin in humidified air at 37 °C with 5% CO_2_. All of these cell lines were authenticated by STR analysis.

### 2.4 Cell transfection

TMEM92‐AS1 cDNA was synthesized and cloned into pcDNA3.1 (Invitrogen, ThermoFisher Scientific, Carlsbad, CA, USA). To knock down or overexpress TMEM92‐AS1 in BGC823 or SGC7901 cells, cells were plated into 6‐well plates, allowed to grow to 60% confluency and transfected with 75 nm si‐TMEM92‐AS1 or pcDNA‐TMEM92‐AS1 using Lipofectamine 2000 (Invitrogen) strictly following the manufacturer’s instructions. SiNC and the empty vector pcDNA were used as negative controls. The transfection efficiencies were detected by qRT‐PCR.

### 2.5 Cell proliferation assays

For MTT assays, transfected BGC823 and SGC7901 cells were seeded into 96‐well plates at a density of 2000 cells per well and were tested with an MTT kit (Sigma, Sigma‐Aldrich, St. Louis, MO, USA) according to the manufacturer’s instructions. For colony formation assays, 1000 transfected cells were placed in each well of 6‐well plates and then cultured in medium containing 10% serum for 2 weeks, and the medium changed every 4 days. The colony spots were fixed with methanol, stained with 0.1% crystal violet (Sigma) and counted. Edu assays were performed with an Edu Cell Proliferation Assay Kit (Ribobio, Cat. No. C10310, Ribibio, Guangzhou, Guangdong, China) according to the manufacturer’s instructions. A higher proportion of red fluorescence indicated a greater number of proliferating cells.

### 2.6 Flow cytometry analysis

The cells were harvested 48 h after transfection. For cell cycle analysis, the cells were stained with propidium iodide (PI) using a CycleTESTTM PLUS DNA Kit (BD Bioscience, Franklin Lakes, NJ, USA) and analysed using flow cytometry (FACScan; BD Biosciences). The proportions of different types of cells in the G0/G1, S and G2/M phases were analysed and compared. For apoptosis analysis, the cells were treated with fluorescein isothiocyanate (FITC)‐Annexin V and propidium iodide (PI) in the dark following the manufacturer’s instructions and were then identified as viable, dead, early apoptotic or late apoptotic cells by FACScan.

### 2.7 Transwell migration and invasion assays

For migration and invasion assays, after transfection, 20 000 cells in 1% FBS medium were placed in the top chamber (8 µm pore size; Millipore, Billerica, MA, USA) with (invasion) or without (migration) a porous pre‐coated membrane, and medium with 10% FBS was added to the bottom chamber. After 24 h, the cells in the top chamber were removed with a cotton swab, and the cells that had migrated through the basement membrane of the chamber were fixed with methanol and stained with crystal violet. The migrated and invasive cells were counted under an IX71‐inverted microscope (Olympus, Tokyo, Japan). The experiments were independently repeated three times.

### 2.8 Western blot assay and antibodies

Cells were lysed with a RIPA lysis solution (Beyotime, Shanghai, China) containing protease inhibitors. Protein lysates were separated by 10% SDS/PAGE electrophoresis, transferred onto 0.22‐mm PVDF membranes (Millipore) and incubated with specific antibodies. The β‐actin antibody (diluted 1/1000) purchased from Abcam was used as a loading control. The anti‐E‐cadherin antibody (diluted: 1/10 000), anti‐vimentin antibody (diluted: 1/2000), anti‐cyclin D1 (diluted: 1/200) and anti‐P19 (diluted: 1/1000) were purchased from Abcam. The anti‐CDK6 antibody (diluted 1/1000) was purchased from Cell Signaling Technology (Danvers, MA, USA). The anti‐MMP9 antibody (diluted 1/1000) and anti‐CCL5 antibody (diluted 1/1000) were purchased from Affinity. The anti‐mouse and anti‐rabbit IgG were purchased from Abcam and diluted to 1/2000 when applied. Autoradiograms were quantified by densitometry (quantity one software; Bio‐Rad, Hercules, CA, USA). imagej software (National Institutes of Health, NIH, Bethesda, MD, USA) was used to calculate the grey value of the western blot band to compare changes in protein levels.

### 2.9 *In vivo* assay

Four‐week‐old athymic male nude mice were purchased from the Animal Centre of the Chinese Academy of Science (Shanghai, China) and were kept under specific pathogen‐free conditions. BGC823 cells were stably transfected with short hairpin (sh)RNA and shCtrl using lentivirus (GeneChem, Shanghai, China). Single‐cell suspensions (2 × 10^6^ in 100 μL) were xenografted subcutaneously into the bilateral hind legs of mice. The tumour volumes were measured as length × width^2^ × 0.5 and recorded every 2 days. Mice were killed after 16 days and the tumours were weighed further study.

### 2.10 Subcellular fractionation location

Separation experiments were performed using the PARIS Kit (Life Technologies) in strict accordance with the manufacturer’s instructions.

### 2.11 Transcriptome sequencing

Total RNA extracted from BGC823 cells with TMEM92‐AS1 knockdown and control cells were quantified and assessed using an Agilent 2200 system (Agilent, Agilent Technologies, Palo Alto, CA, USA). The sequencing library of each RNA sample was prepared using the Ion Proton Total RNA‐Seq Kit v2. The data are presented in Table S2.

### 2.12 RNA immunoprecipitation assays

RNA immunoprecipitation (RIP) experiments were performed with a Magna RIP™ RNA‐Binding Protein Immunoprecipitation Kit (Millipore) following the manufacturer’s instructions. Antibodies for RIP of hnRNPK, YBX1, VDR, KDM4A, DNMT1, LSD1 and BMI1 were purchased from Abcam.

### 2.13 Chromatin immunoprecipitation assays

Chromatin immunoprecipitation (ChIP) assays were performed with the EZ‐CHIP KIT following the manufacturer’s instructions (Cat. 17‐408; Millipore). Antibodies against YBX1 (Cat. ab12148) were purchased from Abcam. The ChIP primer sequences for CCL5 are shown as follows: Forward: CCTAGCCCGGACCAAATTGT, Reverse: CCCACTTCAGTGCTCTGTTC. Quantification of the immunoprecipitated DNA was performed using qPCR. ChIP data were calculated as a percentage relative to the input DNA.

### 2.14 Immunohistochemical assays

Immunohistochemical analysis using CD4 and CD15 antibodies was performed and inflammatory cells quantified.

### 2.15 Statistical analysis

All statistical analyses were performed with spss 17.0 (IBM, SPSS, Chicago, IL, USA). The significance of differences between two different groups was estimated by Student’s *t*‐tests, χ^2^ tests or Wilcoxon tests, as appropriate. The OS rate and DFS rate were calculated by the Kaplan–Meier method with the log‐rank test. The optimal cut‐off values for NWR and NLR were determined by receiver operating curve (ROC) analysis. Univariate and multivariate Cox proportional hazards models were used to screen for independent risk factors affecting prognosis. Variables with *P* < 0.05 according to univariate analysis were recruited into the Cox multivariate regression model for backward:LR analysis. *P* < 0.05 was considered statistically significant. The experiments were all repeated in at least triplicate.

## 3 Results

### 3.1 TMEM92‐AS1 is upregulated in GC tissues and correlated with poor prognosis

**Fig. 1:**
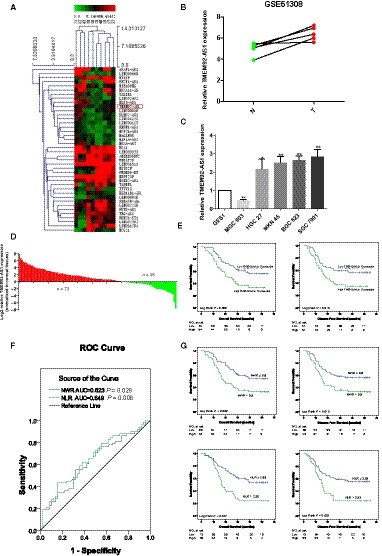
Expression of TMEM92‐AS1 in GC tissues and its clinical significance. (A) Screening TMEM92‐AS1 by bioinformatics analysis. (B) The expression of TMEM92‐AS1 in five pairs of GC tissues and adjacent normal tissues in GEO database. (C) The expression of TMEM92‐AS1 in several GC cell lines; error bars indicate SD. (D) TMEM92‐AS1 was detected in 108 pairs of GC tissues. (E) Patients with high levels of TMEM92‐AS1 expression (*n* = 54) showed reduced OS and DFS compared with patients with low TMEM92‐AS1 expression (*n* = 54) by log‐rang test. (F) Optimal cut‐off values for NWR and NLR were applied with ROC curves. (G) Patients with high NWR (*n* = 48) and NLR (*n* = 75) showed reduced OS and DFS compared with patients with low NWR (*n* = 60) and NLR (*n* = 33) by log‐rank test.

We analysed the GC data (GSE 51308) in the GEO (Gene Expression Omnibus) database and found that the lncRNA TMEM92‐AS1 was upregulated in this group of GC tissues according to lncRNA annotation (Fig. 1A). We performed a statistical analysis of five pairs of tissues in this set of data and found that TMEM92‐AS1 was upregulated in all five pairs of tissues with an average increase of approximately 1.3‐fold (Fig. 1B). Then, we tested the expression levels of TMEM92‐AS1 in several GC cell lines and found that TMEM92‐AS1 was significantly highly expressed in the SGC7901, BGC823, MKN45 and HGC27 cell lines but underexpressed in MGC803 cells (Fig. 1C). This finding suggested that TMEM92‐AS1 may be highly expressed in GC and play some unknown role. We detected the level of TMEM92‐AS1 in 108 paired GC tissues and adjacent normal tissues with qRT‐PCR. TMEM92‐AS1 was upregulated in 73 GC tissues and downregulated in 35 GC tissues compared with their normal counterparts (Fig. 1D).

To investigate the relationships between the expression level of TMEM92‐AS1 and its clinical pathological features, we divided patients into two groups based on TMEM92‐AS1 expression and analysed the clinicopathological features between the high and low groups. The average age of the patients was 60 (31–81) years, the average metastatic lymph node rate (MLNR) was 24.38%, and the average tumour maximum diameter was 6.30 cm. As shown in Table 1, high TMEM92‐AS1 expression was significantly associated with advanced TNM stage and a higher MLNR (both *P* < 0.05). However, some commonly used indicators for evaluating tumours, such as T, N, tumour size and Lauren type, were not found to be associated with TMEM92‐AS1 expression.

**Table 1:** The relationships between clinicopathological factors and TMEM92‐AS1 expression in 108 GC patients.

Variables		TMEM92‐AS1 expression		χ^2^	*P*‐value
		Low	High		
Gender	Male	41	37	0.738	0.39
	Female	13	17		
Age (year)	≤ 60	31	24	1.815	0.178
	> 60	23	30		
T	T1‐2	8	3	2.53	0.112
	T3‐4	46	51		
N	Negative	12	10	0.228	0.633
	Positive	42	44		
TNM	I, II	26	15	4.757	0.029
	III, IV	28	39		
Radicality	R0	44	38	1.824	0.177
	R1, R2	10	16		
MLNR	≤ 24.38%	25	10	8.774	0.003
	> 24.38%	29	42		
Tumour size (cm)	≤ 6.3	33	28	0.942	0.332
	> 6.3	21	26		
CEA (ng·mL^−1^)	≤ 5	45	42	0.532	0.466
	> 5	9	12		
CA199 (µ·mL^−1^)	≤ 37	46	43	0.575	0.448
	> 37	8	11		
Lauren classification	Intestinal	23	18	1.168	0.558
	Diffuse	27	30		
	Mixed	4	6		

To evaluate the relationships between the expression level of TMEM92‐AS1 and the outcomes of patients with GC after gastrectomy, we plotted OS and DFS curves based on the expression levels of TMEM92‐AS1 using Kaplan–Meier analysis and log‐rank tests. Surprisingly, patients with high TMEM92‐AS1 expression exhibited poor OS (*P* = 0.009) and DFS (*P* = 0.019). The median OS and DFS in the high group were 28.9 ± 17.0 and 26.6 ± 17.3 months, respectively, whereas in the low group, they were 40.8 ± 19.1 and 38.3 ± 21.2 months. The 3‐year OS rate comparison was 38.8% vs 63.0% and the 3‐year DFS rate comparison was 37.0% vs 59.3% between the high and low groups, respectively (Fig. 1E).

Our previous study verified the impact of inflammation on the prognosis of cancer patients and the prognostic value of the neutrophil to lymphocyte ratio (NLR) in GC patients [9]. In this study, we used ROC curves to obtain the cut‐off values of the neutrophil to white blood cell ratio (NWR) and the NLR, which were 0.60 and 2.39, respectively (Fig. 1F). Then, we divided patients into high and low groups based on the NWR and NLR cut‐off values and analysed the prognosis of the two groups. Patients with a lower NWR and NLR had better OS and DFS than did patients with a higher NWR and NLR.

To confirm further the prognostic role of TMEM92‐AS1 in GC patients, we performed univariate and multivariate analyses using a Cox proportional hazards regression backward:LR model. Univariate analysis identified 11 prognostic factors (T, N, TNM, radicality, MLNR, tumour size, CEA, CA19‐9, TMEM92‐AS1, NWR and NLR) in OS (Table 2) and 10 prognostic factors (T, N, TNM, radicality, MLNR, tumour size, CEA, TMEM92‐AS1, NWR and NLR) in DFS (Table 3). Multivariate analysis further confirmed that the expression level of TMEM92‐AS1 could be regarded as an independent risk factor for OS in GC patients (*P* = 0.007), along with N (*P* = 0.028), TNM (*P* < 0.001) and tumour size (*P* = 0.006). However, only N (*P* = 0.002) and TNM (*P* < 0.001) were independent risk factors for DFS without the inclusion of the expression level of TMEM92‐AS1.

**Table 2:** Univariate and multivariate analysis of clinicopathological factors for OS.

Variables	Univariate		Multivariate	
	*P*	HB (95% CI)	*P*	HB (95% CI)
Gender	0.926	0.973 (0.540–1.751)		
Age	0.822	0.942 (0.563–1.578)		
T	0.001	2.037 (1.325–3.132)		
N	< 0.001	2.011 (1.610–2.511)	0.028	1.385 (1.035–1.853)
TNM	< 0.001	5.441 (3.363–8.805)	< 0.001	3.309 (1.762–6.211)
Radicality	< 0.001	3.467 (2.013–5.973)		
MLNR	< 0.001	7.321 (3.13–17.124)		
Tumour size	< 0.001	2.784 (1.659–4.675)	0.006	2.226 (1.262–3.924)
CEA	0.044	1.837 (1.017–3.317)		
CA199	0.034	1.922 (1.051–3.512)		
TMEM92‐AS1	0.011	1.990 (1.173–3.375)	0.007	2.150 (1.228–3.766)
NWR	0.008	2.019 (1.201–3.924)		
NLR	0.001	2.386 (1.411–4.034)		

**Table 3:** Univariate and multivariate analysis of clinicopathological factors for DFS.

Variables	Univariate		Multivariate	
	*P*	HB (95% CI)	*P*	HB (95% CI)
Gender	0.984	1.006 (0.559–1.811)		
Age	0.906	0.969 (0.579–1.623)		
T	0.001	2.037 (1.323–3.135)		
N	< 0.001	2.159 (1.707–2.731)	0.002	1.618 (1.197–2.187)
TNM	< 0.001	4.963 (3.100–7.946)	< 0.001	3.015 (1.853–5.485)
Radicality	< 0.001	3.191 (1.853–5.497)		
MLNR	< 0.001	7.303 (3.121–17.086)		
Tumour size	< 0.001	2.530 (1.508–4.245)		
CEA	0.047	1.820 (1.007–3.290)		
CA199	0.079	1.716 (0.903–3.134)		
TMEM92‐AS1	0.022	1.850 (1.091–3.135)		
NWR	0.015	1.898 (1.130–3.188)		
NLR	0.003	2.224 (1.318–3.753)		

### 3.2 TMEM92‐AS1 silencing inhibits GC cell proliferation

**Fig. 2:**
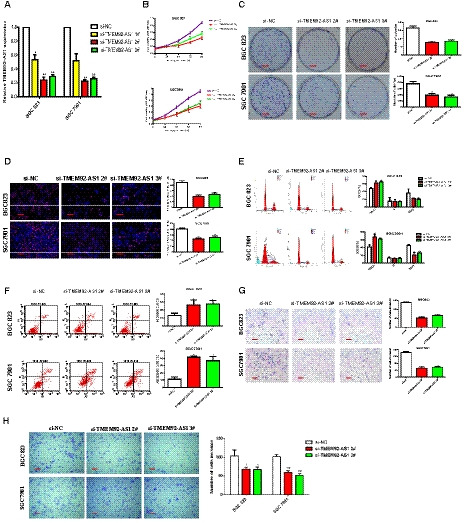
TMEM92‐AS1 regulates GC cell proliferation and migration *in vitro*. (A) Interference efficiency of si‐TMEM92‐AS1 1#, 2# and 3#. (B) MTT assays were performed to detect cell proliferation of BGC823 and SGC7901 after transfection with si‐TMEM92‐AS1 2# and 3#. (C) Colony formation assays of BGC823 and SGC7901 transfected with si‐TMEM92‐AS1 2# and 3# (scale bar: 5 mm). (D) Edu assays of BGC823 and SGC7901 transfected with si‐TMEM92‐AS1 2# and 3# (scale bar: 100 μm). (E,F) 48 h after transfection, cell cycle and apoptosis cell percentage were analysed by flow cytometry. (G,H) Transwell assays were used to detect changes of migration and invasion abilities in BGC823 and SGC7901 cells (scale bar: 100 μm). Student’s *t*‐test was used to compare differences between different groups: *P < 0.05, **P < 0.005. For all of these experiments, values were mean ± SD (error bars) of triplicate samples in representative experiments.

We designed three small interfering (si)RNA (si‐TMEM92‐AS1 1#, 2# and 3#) to detect their interference efficiency and, finally, selected si‐TMEM92‐AS1 2# and 3# for functional experiments in the BGC823 and SGC7901 cell lines (Fig. 2A). Controls were transfected with negative control siRNA (siNC). MTT assays showed that TMEM92‐AS1 knockdown significantly inhibited cell proliferation of BGC823 and SGC7901 cells compared with controls (Fig. 2B). The colony formation assays revealed that clonal survival was significantly decreased when TMEM92‐AS1 was knocked down in BGC823 and SGC7901 cells (Fig. 2C). Edu assays demonstrated that knockdown of TMEM92‐AS1 significantly reduced the percentage of Edu‐positive cells (Fig. 2D).

### 3.3 TMEM92‐AS1 silencing induces cell cycle arrest and apoptosis, and inhibits migration and invasion

To determine whether the effect of TMEM92‐AS1 on proliferation was induced by changing cell cycle progression, we performed flow cytometry analysis. The results showed that TMEM92‐AS1 knockdown led to a significant accumulation of cells in the G0/G1 phase and decreased the proportion of cells in the G2/M phase (Fig. 2E). Through comparison of the proportion of apoptotic cells with the scrambled group, we found that si‐TMEM92‐AS1 2# and 3# transfected cells tended to exhibit higher apoptosis rates (Fig. 2F). Additionally, we used Transwell assays to examine the effect of TMEM92‐AS1 on cell motility, and the results demonstrated that the migratory and invasive abilities of BGC823 and SGC7901 cells transfected with si‐TMEM92‐AS1 2# and 3# were obviously attenuated (Fig. 2G,H).

**Fig. 3:**
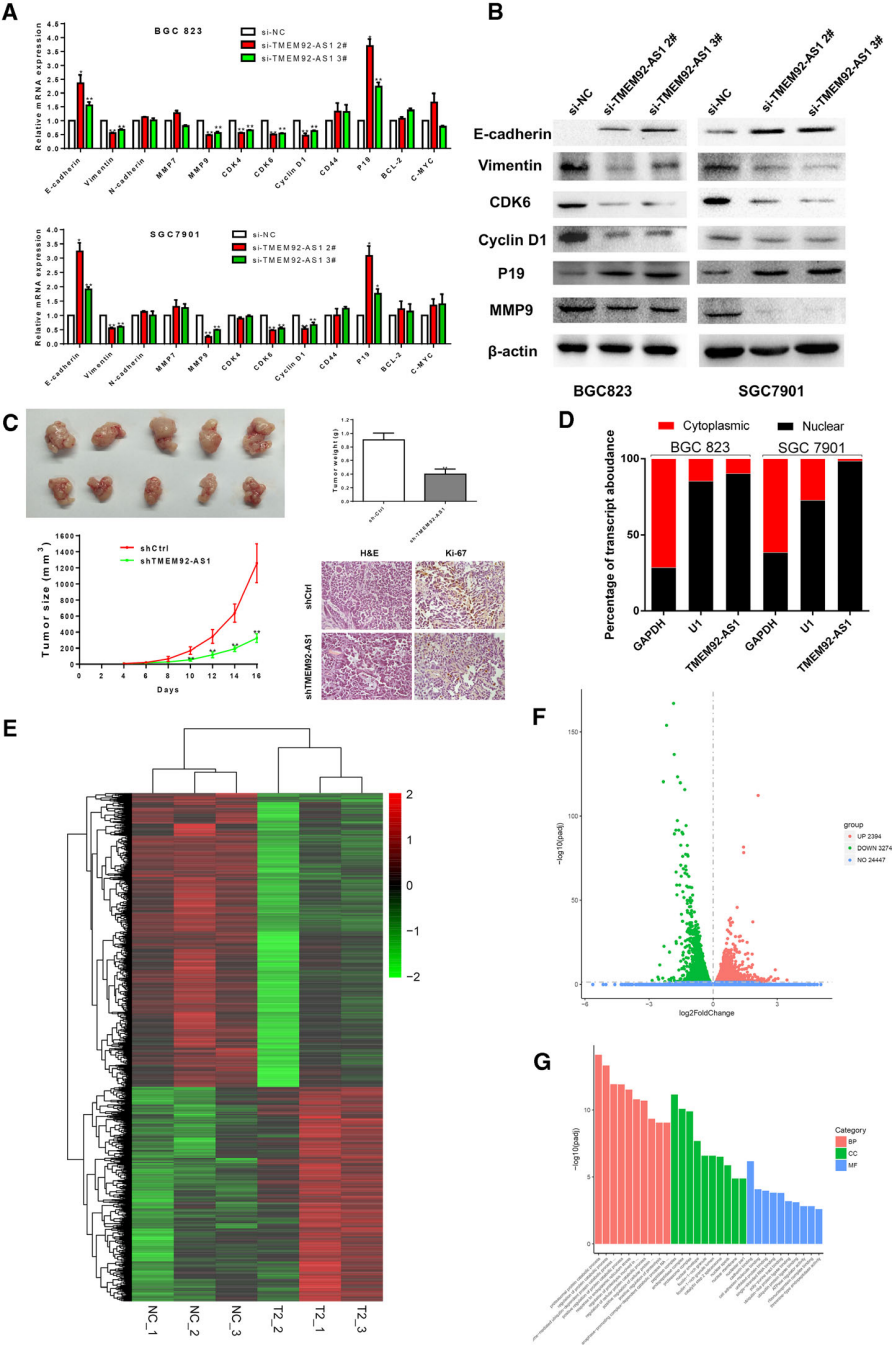
TMEM92‐AS1 regulates expression of cellular functional genes. (A B) RT‐qPCR and western blot assays were used to detect expression changes of cellular functional genes and proteins. (C) ShCtrl and shTMEM92‐AS1 were stably transfected into BGC823 cells and then injected into subcutaneous of nude mice. Tumour volumes were measured every 2 days after macroscopic formation of subcutaneous tumours (scale bar: 100 μm). (D) Nuclear and cytosolic separation assays were used to detect distribution of TMEM92‐AS1 in BGC823 and SGC7901 cells, GAPDH was used as a cytoplasmic marker and U1 was used as a nuclear marker. (E) Transcriptome sequencing results of gene transcripts altered (> 1.0‐fold) after knockdown of TMEM92‐AS1 by si‐TMEM92‐AS1 2# in BGC823 cells. (F) Number of genetic changes found by transcriptome sequencing. (G) Gene ontology analysis of all the genes altered after TMEM92‐AS1 knockdown. *P < 0.05, **P < 0.005 by Student’s *t*‐test. For all of these experiments, values were mean ± SD (error bars) of triplicate samples in representative experiments.

Then, we detected the expression of several functionally related genes at the cellular level. We found that the mRNA levels of E‐cadherin and P19 were significantly upregulated, whereas those of vimentin, CDK6, cyclin D1 and MMP9 were significantly decreased in BGC823 and SGC7901 cells transfected with si‐TMEM92‐AS1 2# and 3# (Fig. 3A). We used western blot assays to detect the expression level of these proteins and found that the trends of these protein levels were completely consistent with their mRNA levels (Fig. 3B). This finding indicates that TMEM92‐AS1 is indeed influential in epithelial‐mesenchymal transition (EMT), proliferation and invasion of GC cells.

### 3.4 TMEM92‐AS1 regulates GC cell proliferation *in vivo*

To further explore whether TMEM92‐AS1 accelerates tumorigenesis *in vivo*, BGC823 cells stably transfected with shTMEM92‐AS1 or shCtrl were inoculated into nude mice. All nude mice formed subcutaneous xenograft tumours. Sixteen days after injection, the tumours in the shTMEM92‐AS1 group were substantially smaller than those in the shCtrl group (Fig. 3C). Moreover, when the tumours were weighed at the end of the experiment, we found that the average weight of the shTMEM92‐AS1 group was significantly lower than that of the shCtrl group. The tumours developed from stably shTMEM92‐AS1‐transfected mice displayed a decreased positive rate of Ki‐67 relative to the shCtrl group (34.65 ± 2.95% vs 11.41 ± 1.86% , *P* < 0.05 ). These results indicated that knockdown of TMEM92‐AS1 could inhibit the tumorigenic ability of GC cells and tumour progression.

### 3.5 TMEM92‐AS1 functions by regulating the downstream target gene CCL5

**Fig. 4:**
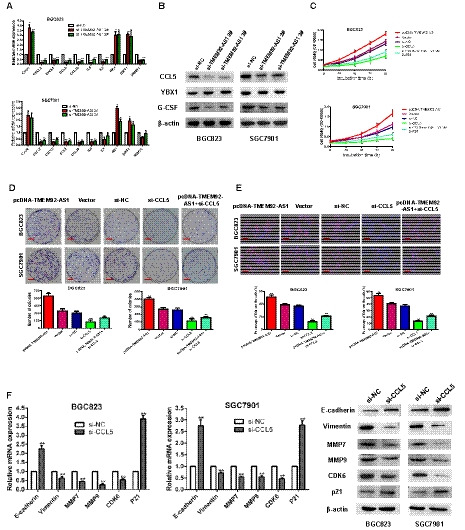
CCL5 was confirmed as a downstream target gene of TMEM92‐AS1. (A,B) RT‐qPCR and western blot were used to verify changed genes and proteins after TMEM92‐AS1 knockdown. (C) MTT assays were performed to detect the effect of knockdown of CCL5 and overexpression of TMEM92‐AS1 on cell proliferation. (D) Colony formation assays of knockdown of CCL5 and overexpression of TMEM92‐AS1 in BGC823 and SGC7901 cells (scale bar: 5 mm). (E) Edu assays of knockdown of CCL5 and overexpression of TMEM92‐AS1 in BGC823 and SGC7901 cells (scale bar: 100 μm). (F) RT‐qPCR and western blot were used to detect changes in TMEM92‐AS1‐related functional genes expression after knockdown of CCL5. *P < 0.05, **P < 0.005 by Student’s *t*‐test. For all of these experiments, values were mean ± SD (error bars) of triplicate samples in representative experiments.

To explore the mechanism by which TMEM92‐AS1 regulates the function of GC cells, we verified the localization of TMEM92‐AS1 by subcellular separation and localization analysis and found that it was especially high in the nucleus (Fig. 3D). This finding suggests that TMEM92‐AS1 may play a major role in transcription. To identify downstream target genes regulated by TMEM92‐AS1, we performed RNA transcriptome sequencing, comparing the control to si‐TMEM92‐AS1 2#. A total of 3274 mRNA showed decreased abundance (179 ≤ 1.0‐fold), and silencing TMEM92‐AS1 increased the abundance of 2394 mRNA (283 ≥ 1.0‐fold) (Fig. 3E,F). Gene oncology analysis showed that the most representative biological processes included pathways involved in cellular protein catabolic process, cell adhesion and cell metabolism (Fig. 3G). From the sequencing results, we selected some inflammation‐related and functional genes to verify their expression in BGC823 and SGC7901 cells. The qRT‐PCR results showed that C‐X‐C motif chemokine ligand 2 (CXCL2), CXCL3, C‐C motif chemokine ligand 5 (CCL5) and CCL20 were significantly downregulated, whereas C‐X‐C motif chemokine receptor 2 (CXCR2), RB1 and sirtuin 1 (SIRT1) were upregulated when TMEM92‐AS1 was knocked down by si‐TMEM92‐AS1 2# and 3# in both BGC823 and SGC7901 cells (Fig. 4A). Interleukin 6 (IL‐6) and IL‐8 were downregulated in BGC823 cells and upregulated in SGC7901 cells when TMEM92‐AS1 was knocked down. DNA methyltransferase 1 (DNMT1) was downregulated in BGC823 but its expression trend was not significant in SGC7901.

By consulting a large body of literature, we identified the CCL5 gene and found that it was closely related to the occurrence and progression of many tumours. Gao *et al*. [10] reported that CCL5 interacts with its unique receptor CCR5 in the tumour microenvironment, which affects the metabolic activity of breast cancer cells, thereby promoting cell tumorigenesis and progression during breast cancer onset. Chang *et al*. [11] reported that knockdown of CCL5 delayed tumour growth and reduced CD8^+^ T‐cell apoptosis, whereas CCL5/CCR5 signalling recruited T regulatory cells to the tumour to kill antitumour CD8^+^ T cells, contributing to the immune escape of colorectal cancer. In view of these findings, we selected CCL5 as a possible downstream target gene of TMEM92‐AS1. Western blot assays confirmed that the expression of the CCL5 protein was decreased after knockdown of TMEM92‐AS1 in both BGC823 and SGC7901 cells (Fig. 4B).

We designed si‐CCL5 and pcDNA‐TMEM92‐AS1 to verify the effects of TMEM92‐AS1 and CCL5 on cell function. MTT assays verified that overexpression of TMEM92‐AS1 can promote the proliferation of gastric cancer cells, knockdown of CCL5 can inhibit proliferation, and overexpression of TMEM92‐AS1 can accelerate the sluggish proliferation rate caused by knockdown of CCL5 (Fig. 4C). Colony formation assays demonstrated that clonal survival was enhanced when overexpressing TMEM92‐AS1 and reduced when CCL5 was knocked down, and overexpression of TMEM92‐AS1 corrected the clonal survival delayed by CCL5 knockdown (Fig. 4D). Edu assays indicated that the percentage of Edu‐positive cells was increased with TMEM92‐AS1 overexpression and decreased with CCL5 knockdown, and overexpression of TMEM92‐AS1 reversed the decrease in the percentage of Edu‐positive cells caused by the knockdown of CCL5 (Fig. 4E). All these results were confirmed in both BGC823 and SGC7901 cells.

Next, we examined the effect of CCL5 knockdown on the expression of some functional genes affected by TMEM92‐AS1. The qPCR results verified that the mRNA of E‐cadherin and P21 were upregulated with CCL5 knockdown, whereas vimentin, MMP7, MMP9 and CDK6 were downregulated. Western blot assays showed the same trend at the protein level (Fig. 4F).

### 3.6 TMEM92‐AS1 interacts with YBX1

We tested the expression level of TMEM92 after knocking down TMEM92‐AS1 and found that the expression level of TMEM92 was not affected by knockdown of TMEM92‐AS1. Considering that TMEM92‐AS1 is mainly located in the nucleus rather than the cytoplasm, suggesting that TMEM92‐AS1 acts primarily at the transcriptional level, it may interact with molecules or proteins in the nucleus. Numerous studies have shown that most lncRNA function by interacting with RNA‐binding proteins (RBP) [12, 13, 14]. To explore the precise functional mechanism of TMEM92‐AS1, we tried to identify the RBP that bind to it. Zhang *et al*. [15] reported that the RBP Y‐box binding protein 1 (YBX1), which is a transcription factor and has a tumour‐promoting effect, can interact with the lncRNA HOSC‐AS3. Salifou *et al*. [16] reported that lysine demethylase 4A (KDM4A) can interact with RNA Polymerase I, which is associated with ribosomal RNA and increases rDNA transcription. Tang *et al*. [17] reported that lncRNA‐OG interacts with the heterogeneous nuclear ribonucleoprotein K (hnRNPK) protein to activate the bone morphogenetic protein signalling pathway, and that hnRNPK can activate lncRNA‐OG transcription.

**Fig. 5:**
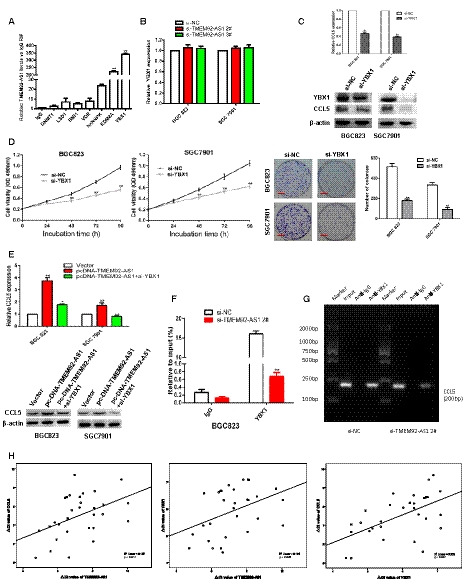
YBX1 acted as a RBP for TMEM92‐AS1. (A) RIP experiments were performed for YBX1/KDM4A/hnRNPK/VDR/BMI1/LSD and DNMT1 and the coprecipitated RNA was performed by qRT‐PCR for TMEM92‐AS1. (B) RT‐qPCR was used to detect YBX1 mRNA level after TMEM92‐AS1 knockdown. (C) RT‐qPCR and western blot were performed to detect CCL5 expressions after YBX1 knockdown. (D) MTT and colony formation assays were performed to verify the effect of YBX1 on cell proliferation. (E) RT‐qPCR and western blot were performed to detect the effect of TMEM92‐AS1 overexpression and YBX1 knockdown on CCL5 expression. (F,G) ChIP results of YBX1 of the promoter region of CCL5 after si‐TMEM92‐AS1 2# in BGC823 cell. (H) The expression of TMEM92‐AS1, YBX1 and CCL5 was detected in 30 GC tissues and the expressions of these three were positively correlated. *P < 0.05, **P < 0.005 by Student’s *t*‐test. For all of these experiments, values were mean ± SD (error bars) of triplicate samples in representative experiments.

We performed RNA immunoprecipitation of the lncRNA‐RBP complex using antibodies against YBX1, KDM4A, hnRNPK and other proteins to measure the amount of TMEM92‐AS1 interacting with RBP immunoprecipitates. As shown in Fig. 5A, the abundance of TMEM92‐AS1 that bound to YBX1 was the highest, exceeding that of KDM4A, hnRNPK and the other proteins.

Next, we examined the effect of TMEM92‐AS1 on YBX1 expression. The qPCR results showed that the transcription level of YBX1 mRNA was not affected (Fig. 5B). Western blot assays confirmed the qPCR results (Fig. 4B). We next verified the effect of YBX1 on CCL5 expression and found that CCL5 expression was downregulated after YBX1 knockdown at both the mRNA and protein levels (Fig. 5C).

Zhou *et al*. [18] reported that knockdown of YBX1 inhibited migration and EMT in nasopharyngeal carcinoma. Lim *et al*. [19] reported that YBX1 acted as an oncogene in bladder cancer and was expected to become a therapeutic target for bladder cancer patients. In this study, we examined the effect of YBX1 on gastric cancer cells and found that knockdown of YBX1 significantly inhibited the proliferation and clonal survival of both BGC823 and SGC7901 cells (Fig. 5D).

Next, we detected the level of CCL5 expression with synchronous TMEM92‐AS1 overexpression and YBX1 knockdown. The qPCR assays showed that TMEM92‐AS1 overexpression significantly upregulated CCL5 mRNA, and that CCL5 mRNA expression with TMEM92‐AS1 overexpression accompanied by YBX1 knockdown was higher than that of YBX1 knockdown alone (Fig. 5E). An identical trend at the protein level was also confirmed by western blot analysis.

Considering that YBX1 may regulate the transcription of target genes by binding to their promoter regions, we examined whether knockdown of TMEM92‐AS1 affected YBX1 binding to the promoter region of CCL5. ChIP assays followed by qPCR confirmed that knockdown of TMEM92‐AS1 significantly decreased YBX1 binding to the CCL5 promoter region (Fig. 5F,G). Then, we used qPCR to detect the expression of TMEM92‐AS1, YBX1 and CCL5 in 30 GC tissues and found that the expression levels of TMEM92‐AS1, YBX1 and CCL5 were positively correlated (Fig. 5H). These results demonstrated that TMEM92‐AS1 functions, at least in part, by binding to YBX1 to affect the expression of the downstream target gene CCL5.

### 3.7 TMEM92‐AS1 affects inflammatory indicators

**Fig. 6:**
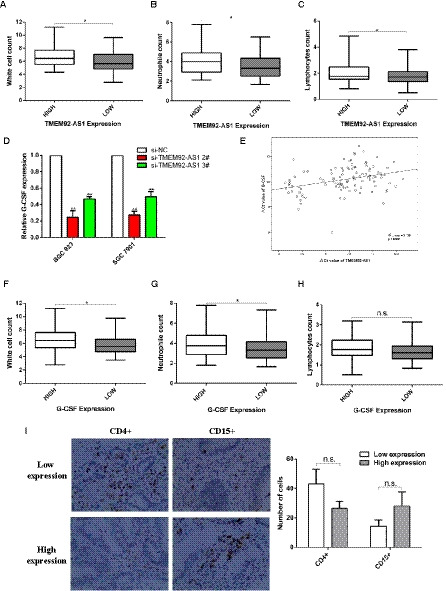
Relationships between TMEM92‐AS1 and G‐CSF expression levels with leucocyte counts. (A–C) Relationships between TMEM92‐AS1 expression and white cell, neutrophil and lymphocyte counts (*n* = 108). (D) Expression levels of G‐CSF after TMEM92‐AS1 knockdown in BGC823 and SGC7901 cells. (E) The level of TMEM92‐AS1 in GC tissues was significantly correlated with that of G‐CSF (*n* = 108); (F–H) Relationships between G‐CSF expression and white cell, neutrophil and lymphocyte counts (*n* = 108). (I) Comparison of infiltrating CD4^+^ lymphocytes and CD15^+^ neutrophils in GC tissues in different patients (*n* = 20, scale bar: 100 μm). *P < 0.05, n.s. = not significant by Student’s *t*‐test. For all of these experiments, values were mean ± SD (error bars) of triplicate samples in representative experiments.

We have already mentioned that NWR and NLR can predict the prognosis of patients with GC, and we next explored whether TMEM92‐AS1 was related to white cells, neutrophils and lymphocytes. We compared the levels of white cells, neutrophils and lymphocytes in the TMEM92‐AS1 high and low expression groups and found that there were significant differences between the two groups in these three cells. The TMEM92‐AS1 high expression group had higher white cell, neutrophil and lymphocyte counts than the TMEM92‐AS1 low expression group (Fig. 6A–C). Granulocyte colony‐stimulating factor (G‐CSF) is widely used in chemotherapy patients to increase the production of granulocytes. We suspected that TMEM92‐AS1 might affect the counts of white cells, neutrophils and lymphocytes by regulating the expression of G‐CSF. We examined the expression level of G‐CSF after knockdown of TMEM92‐AS1 and found that G‐CSF was downregulated in both BGC823 and SGC7901 cells (Figs 4B and 6D). Then, we examined the expression levels of TMEM92‐AS1 and G‐CSF in GC tissues and found that the expression of TMEM92‐AS1 and G‐CSF were positively correlated (Fig. 6E). Finally, we compared the white cell, neutrophil and lymphocyte counts in the G‐CSF high and low expression groups and found that the G‐CSF high expression group had more white blood cells and neutrophils (Fig. 6F,G) but that there was no significant difference in lymphocyte counts (Fig. 6H). In addition, we detected the expression level of TMEM92‐AS1 in GC tissue, the level of G‐CSF in peripheral blood, and the infiltrating CD15^+^ neutrophils and CD4^+^ T lymphocytes in 20 patients. We found that there were more CD4^+^ T lymphocytes and fewer CD15^+^ neutrophils in tumour tissues with low TMEM92‐AS1 expression. Although the differences were not statistically significant, the trends were quite obvious. Because of the extremely low level of G‐CSF in serum, it was difficult to detect the level of G‐CSF in about half of the patients. Unfortunately, our ELISAs did not find significant differences in G‐CSF in patients with high and low tm expression.

## 4 Discussion

Before the discovery of multifunctional non‐coding (nc)RNA, studies of gene function were limited to protein‐coding genes because the proteins encoded by these genes were thought to be the main enablers of function. However, with the development of high‐throughput sequencing technology and the deepening of ncRNA functional research, it was gradually found that ncRNA were also important regulatory genes of cell function, especially in tumours. Currently, ncRNA are classified as active biological functional molecules rather than ‘transcriptional noise’ [20, 21, 22]. Moreover, a large amount of research results has revealed the important functions of the latest reported lncRNA as oncogenes or tumour suppressor genes in different types of tumours [3, 23, 24].

In our present study, we found that the expression level of TMEM92‐AS1 in GC tissues was significantly higher than that in corresponding nontumour tissues. Higher expression of TMEM92‐AS1 was positively associated with advanced TNM stages and higher MLNR. Moreover, higher expression of TMEM92‐AS1 indicated poor OS and DFS in patients with GC and could be regarded as an independent risk factor for OS. Combined with the gain and loss‐of‐function results, we found that TMEM92‐AS1 promoted GC cell proliferation and migration and inhibited apoptosis. These results further revealed that TMEM92‐AS1 may play an oncogene role in the development and progression of GC.

TMEM92‐AS1, which is located on chromosome 17q21.33, is an antisense transcript of TMEM92. To date, no researchers have reported on the function of TMEM92‐AS1. LncRNA research in neoplastic disease should focus on exploring its various mechanisms of affecting tumour cell functions. Zhuo *et al*. [25] reported that the lncRNA GMAN binds competitively to GMAN‐AS to regulate ephrin A1 translation. Jiang *et al*. [26] reported that the lncRNA CCAT1 forms a complex with TP63 and SOX2 and then binds to the super enhancer of EGFR, thereby promoting the expression of EGFR and activating the MEK/ERK1/2 and PI3K/AKT signalling pathways.

To decipher the precise mechanisms of TMEM92‐AS1, RNA‐Seq, subcellular fractionation location assays and RIP and ChIP assays were performed. Our study first reported the carcinogenic and cancer‐promoting effects of TMEM92‐AS1 in GC and showed that TMEM92‐AS1 regulated the expression of CCL5 by binding to YBX1 to exert function. YBX1 is a cold‐shock domain protein and is involved in the transcription and translational regulation of many genes. Zhang *et al*. [27] reported that increased YBX1 expression was significantly associated with tumour differentiation status, size and lymph node metastasis and predicted poor survival of many solid tumours. Yang *et al*. [28] reported that micro (mi)R‐S8 can suppress the stemness of human melanoma stem‐like cells by regulating the expression of transcription factor YBX1.

In our study, we found that YBX1, as a transcription factor and RBP, can regulate the expression of CCL5 and that knockdown of YBX1 inhibited the proliferation and colonization of GC cells. TMEM92‐AS1 exerts its powerful and diverse biological functions through YBX1. It has been observed that nuclear lncRNA are involved in chromatin interactions and transcriptional regulation by binding to DNA and RNA, and cytoplasmic lncRNA regulates transcription and translation and participates in cell signalling pathways [29, 30, 31]. Unfortunately, we did not explore the mechanisms of TMEM92‐AS1 function separately in the nucleus and cytoplasm.

The complicated and diverse interactions between tumour cells and the microenvironment, especially immune cells, have important influences on their biological behaviour [32, 33, 34]. Lnc‐DC was the first lncRNA proven to be immune‐related. Wang found that lnc‐DC was exclusively expressed in human conventional dendritic cells and that knockdown of lnc‐DC impaired their differentiation from monocyte and bone marrow cells to DC [35]. Luo discovered hematopoietic stem cell (HSC)‐specific lncRNA (lncHSC) by sequencing and found that knocking down lncHSC strongly affected HSC self‐renewal and lineage commitment [36].

In our study, we accidentally discovered that the lncRNA TMEM92‐AS1 was related to the white cell, neutrophil and lymphocyte counts. We tried to explore the reasons for this phenomenon and speculated that there may be colony‐stimulating factors (CSF) involved in this role. It was reported in the literature that G‐CSF is highly produced by stromal myofibroblasts and carcinoma cells in GC tissues [37]. Gene expression correlation detection confirmed the correlation between TMEM92‐AS1 and G‐CSF, and G‐CSF seems to be regulated by TMEM92‐AS1. To the best of our knowledge, it has not been previously reported that G‐CSF produced by tumour tissues can be regulated by lncRNA, and G‐CSF can affect blood cell counts in patients. Our study demonstrated for the first time that G‐CSF produced in gastric cancer tissue is upregulated by highly expressed TMEM92‐AS1 and that higher expression of G‐CSF is associated with increased peripheral blood leucocytes and neutrophil counts. Unfortunately, we did not retain blood samples from all of the patients, so we were unable to detect the level of TMEM92‐AS1 and G‐CSF in patients’ serum or explore the correlations between their expression in serum.

## 5 Conclusions

In summary, we have revealed a novel lncRNA, TMEM92‐AS1, which has potential for use as a biomarker to predict OS and DFS in GC patients, and which has oncogenic properties by promoting the proliferation and migration of GC cells. In addition, we discovered that TMEM92‐AS1 impacts GC carcinogenesis partially through binding with YBX1 to regulate the expression of CCL5. Further investigation is required to clarify the underlying mechanism by which TMEM92‐AS1 regulates G‐CSF to affect peripheral leucocyte counts and to determine how it regulates infiltrating immune cells in midstream tissues.

## Conflict of interest

The authors declare no conflict of interest.

## Author contributions

SS, XH and JW performed this experiments, and analysed and interpreted the data. SS, ZY, YL and ZZ prepared the original draft. YX and DM initialled the project. HS and YW collected the clinical pathological data, prepared supplementary experiments and revised the manuscript. All authors have read and accepted this manuscript.

## Supporting information


**Table S1.** Sequences of primers used in QPCR experiments.

**Table S2.** Transcriptome sequencing results of knockdown TMEM92‐AS1 compared with the siNC group from the BGC823 cells.
