# Supplementary material for: A novel long noncoding RNA, TMEM92‐AS1, promotes gastric cancer progression by binding to YBX1 to mediate CCL5
Source: Mol Oncol. 2021 Jan 12;15(4):1256–73. doi: 10.1002/1878-0261.12863 (PMC8024739; doi:10.1002/1878-0261.12863)
Supplement: Supplementary file 1 — Table S1. Sequences of primers used in QPCR experiments. [file MOL2-15-1256-s001.docx]

| Gene | Forward | Backward |
| --- | --- | --- |
| TMEM92-AS1 | GCTGCTACAGTTTCCCGTA | GCCCCAAGAGTGGGATGTAG |
| GAPDH | GGGAGCCAAAAGGGTCAT | GAGTCCTTCCACGATACCAA |
| P21 | TGTCCGTCAGAACCCATGC | AAAGTCGAAGTTCCATCGCTC |
| P63 | GTCATTTGATTCGAGTAGAGGG | CTGGGGTGGCTCATAAGGT |
| TMEM92 | GCCTGCCCCAAAGGATTCAA | CAAAAGACGGACAGGATGACC |
| DNMT1 | AGAACGGTGCTCATGCTTACA | CTCTACGGGCTTCACTTCTTG |
| RB | CTCTCGTCAGGCTTGAGTTTG | GACATCTCATCTAGGTCAACTGC |
| BMF | GAGCCATCTCAGTGTGTGGAG | GCCAGCATTGCCATAAAAGAGC |
| CXCL3 | AACCGAAGTCATAGCCACAC | TGCTCCCCTTGTTCAGTATC |
| CXCL8 | TTTTGCCAAGGAGTGCTAAAGA | AACCCTCTGCACCCAGTTTTC |
| CCL5 | CCAGCAGTCGTCTTTGTCAC | CTCTGGGTTGGCACACACTT |
| MMP9 | AACCAATCTCACCGACAG | AAAGGCGTCGTCAATCAC |
| CDK4 | CATGTAGACCAGGACCTAAGG | GGAGGTCGGTACCAGAGTG |
| CDK6 | TGCACAGTGTCACGAACAGA | ACCTCGGAGAAGCTGAAACA |
| IL6 | ATGTAGCCGCCCCACACAGA | CATCCATCTTTTTCAGCCAT |
| G-CSF | GCTGCTTGAGCCAACTCCATA | GAACGCGGTACGACACCTC |
| MMP7 | GAGATTTTATCTCTATGACTCAG | CCTTTGACACTAATCGATC |
| CCL20 | GCAAGCAACTTTGACTGCTG | CAAGTCCAGTGAGGCACAAA |
| E-cadherin | CGAGAGCTACACGTTCACGG | GGGTGTCGAGGGAAAAATAGG |
| N-cadherin | TCAGGCGTCTGTAGAGGCTT | ATGCACATCCTTCGATAAGACTG |
| Vimentin | GACGCCATCAACACCGAGTT | CTTTGTCGTTGGTTAGCTGGT |
| Cyclin D1 | GCTGCGAAGTGGAAACCATC | CCTCCTTCTGCACACATTTGAA |
| Casp3 | CATGGAAGCGAATCAATGGACT | CTGTACCAGACCGAGATGTCA |
| Casp9 | CTGTCTACGGCACAGATGGAT | GGGACTCGTCTTCAGGGGAA |
| Bcl2 | GGTGGGGTCATGTGTGTGG | CGGTTCAGGTACTCAGTCATCC |
| CD44 | CTGCCGCTTTGCAGGTGTA | CATTGTGGGCAAGGTGCTATT |
